# Relationship between hospitalization from cannabis usage and pulmonary tuberculosis in Thailand from 2017 to 2022

**DOI:** 10.1371/journal.pone.0312139

**Published:** 2024-12-05

**Authors:** Kemmapon Chumchuen, Wit Wichaidit, Virasakdi Chongsuvivatwong

**Affiliations:** Department of Epidemiology, Faculty of Medicine, Prince of Songkla University, Hatyai, Songkhla, Thailand; Seth GS Medical College and KEM Hospital: King Edward Memorial Hospital and Seth Gordhandas Sunderdas Medical College, INDIA

## Abstract

In June 2022, Thailand legalized recreational cannabis. Currently, cannabis is now the most consumed drug. Cannabis usage can increase inflammatory responses in the respiratory tract. Sharing of cannabis waterpipes has been linked to increased tuberculosis risks. Using a national in-patient databank, we aimed to 1) describe the spatiotemporal correlation between cannabis-related and tuberculosis hospital admissions, and 2) compare the rate of subsequent pulmonary tuberculosis admission between those with prior admissions for cannabis-related causes and those without. Both admission types were aggregated to the number of admissions in monthly and provincial units. Temporal and spatial patterns were visualized using line plots and choropleth maps, respectively. A matched cohort analysis was conducted to compare the incidence density rate of subsequent tuberculosis admission and the hazard ratio. Throughout 2017–2022, we observed a gradual decline in tuberculosis admissions, in contrast to the increase in cannabis-related admissions. Both admissions shared a hotspot in Northeastern Thailand. Between matched cohorts of 6,773 in-patients, the incidence density rate per 100,000 person–years of subsequent tuberculosis admissions was 267.6 and 165.9 in in-patients with and without past cannabis-admission, respectively. After adjusting for covariates, we found that a cannabis-related admission history was associated with a hazard ratio of 1.48 (P = 0.268) for subsequent tuberculosis admission. Our findings failed to support the evidence that cannabis consumption increased pulmonary tuberculosis risk. Other study types are needed to further assess the association between cannabis consumption and pulmonary tuberculosis.

## Introduction

Pulmonary tuberculosis (TB) is a disease caused by *Mycobacterium tuberculosis* (MTB). Once the disease is active, prolonged treatment is necessary [1]. Since the discovery of MTB in 1882, several factors have been reported to increase TB risk. Demographic risk factors include male sex [2] and living in low socioeconomic areas with poor hygiene [3, 4] and high TB prevalence. Underlying diseases reported as TB risk factors include diabetes mellitus [5] and human immunodeficiency virus (HIV) infection [6, 7]. Behavioral TB risk factors include smoking [8–10] and sharing instruments used for substances such as tobacco and cannabis [11, 12].

After the ban on opium, cannabis has become the most widely consumed and cultivated psychoactive drug for recreational and medicinal reasons worldwide. Currently, cannabis users are estimated to comprise 2.5% of the global population [13]. In 2022, cannabis was legalized in Thailand, as a result of a political campaign to use cannabis as a traditional herb to stimulate the domestic economy [14].

Several health risks have been reported from cannabis usage, including increased inflammatory responses in the respiratory system, leading to conditions such as chronic cough, chronic bronchitis, and chronic obstructive pulmonary disease [15, 16]. Pulmonary infections have also been reported to result from cannabis smoking. A case of necrotizing pneumonia originating from water contaminated with *Pseudomonas aeruginosa* in a cannabis water pipe has been reported [17].

Regarding the risk of TB from cannabis usage, a contract tracing study in Australia reported an increased risk of acquiring TB among individuals who shared water pipes with a TB index case, with the risk comparable to that observed among household contacts of patients with TB [18]. This highlighted shared substance usage as a common route of pathogen transmission. French et al. conducted a systematic review on the association between cannabis consumption and TB, comprising studies both with and without a comparison group. However, the studies with comparison groups either focused on latent TB infection or reported no association between cannabis usage and active pulmonary TB after adjusting for confounders [19]. Apart from shared cannabis smoking instruments, the potential impact of cannabis-induced inflammation on the severity of active TB development in at-risk populations remains unexplored.

To date, no systematic investigation has estimated the extent of the linkage between cannabis usage and pulmonary TB in low- and middle-income countries such as Thailand, where both issues are highly prevalent. Data mining could serve as a valuable initial approach to explore the connection between hospitalization for cannabis use and subsequent hospitalization for TB.

The Thai Health Information Portal (THIP) is a national in-patient open data warehouse established through collaborations among the National Science and Technology Development Agency (NSTDA), National Health Security Office (NSHO), and the Prince of Songkla University. THIP contains nationwide admission information of Thai inpatients who have filed for reimbursement from the NHSO for medical fees. In-patient data from 2017 to 2022 have been made available for analysis [20, 21]. Therefore, using the THIP data, the study objectives were: 1) to assess the spatiotemporal correlation between cannabis-related hospital admissions and subsequent TB-related hospital admissions, and 2) to compare the incidence density rate of subsequent pulmonary TB hospital admission between in-patients previously diagnosed with cannabis-related conditions and those without such diagnoses.

## Material and methods

### Data source

This study was conducted using the THIP data warehouse, which comprised anonymized admission records of Thai in-patients admitted between 2017 and 2022.

### Data curation

THIP data were queried using the International Classification of Diseases (ICD-10) codes. Admission records of in-patients with conditions matching the queried ICD-10 codes as either their primary or secondary diagnosis were selected for data extraction. As a proxy for cannabis user population, individuals admitted with conditions including mental and behavioral disorders due to use of cannabinoids, cannabis poisoning, and cannabinosis were queried from THIP [22, 23]. To obtain the pulmonary TB history of these in-patients, ICD-10 codes related to TB were also used in the query processes. All ICD-10 codes utilized in this process, along with their descriptions, are detailed in **S1 Dataset**.

### Variables

Both the exposure and outcome variables were derived from the full diagnoses of the in-patients. In THIP, each admission record contains one primary diagnosis column and 21 columns for secondary diagnoses. For the exposure variable, we defined in-patients with cannabis-related conditions (**S1 Dataset**) listed in any diagnosis column as having a history of cannabis-related admission. A similar definition was employed for the outcome variable, which was pulmonary TB admission. Both the exposure and outcome variables were treated as binary variables in this study.

Other variables available in the databank included in this study were age, sex, province of residence, and date of admission. The date of admission was used to determine the causal relationship between the exposure and outcome variables. Age was categorized into two groups: <40 years and ≥ 40 years. For the first objective, co-diagnoses were pre-selected for estimating increased risk as a hazard ratio (HR). We selected common (>10%) co-diagnoses presented in the first admission in any diagnosis column of those who were later admitted with pulmonary TB.

### Statistical analysis

For the first objective, we aggregated admission counts into monthly units and plotted a line graph to display the temporal patterns over the years. For spatial patterns, we aggregated the admission counts into province-level units for each year. The counts were subsequently divided by the population of each province and presented as the incidence rate (IR) per 100,000 population. To visualize the spatial patterns, we plotted choropleth maps of the IRs for both admission types from 2017 to 2022.

Regarding the second objective, we matched in-patients admitted for cannabis-related causes with general in-patients from the THIP data based on selected variables, including age, sex, year of admission and superdistrict. We calculated the incidence density rate (IDR) by dividing the number of subsequent TB admissions in each group by the cumulative person–years. The person–year was defined as the time between the patient’s first admission entry in the THIP data and their endpoint, which was either the last admission entry if pronounced dead or the 30^th^ of September 2022, which was the last date of entry of THIP data for alive in-patients.

We then statistically compared the matched populations using univariate analysis to assess the matching balance and differences in demographic and outcome-related variables. The pre-selected common co-diagnoses from those who later developed subsequent TB admission were also included in this univariate process. We used the Mann–Whitney *U* test, chi-squared test, and Poisson Exact test to assess non-normally distributed continuous variables, categorical variables, and comparison between IDRs, respectively.

To assess the increased risk of subsequent pulmonary TB admission while adjusting for covariates, we performed a Cox regression between the matched populations for the outcome of the first subsequent TB admission. We displayed the result of the Cox regression as an adjusted hazard ratio (HR) table.

*P<*0.05 was considered statistically significant in all performed analyses. Estimations of increased TB risk (HR) were accompanied by 95% confidence intervals (CI). All data were managed and analyzed using R program version 4.2.3 [24].

### Ethical approval

This study was approved by the Human Research Ethics Committee of the Faculty of Medicine, Prince of Songkla University (REC 66-479-18-5). As this study was conducted using anonymized secondary data, the need for informed consent was waived.

## Results

### Spatiotemporal relationships

**Fig 1:**
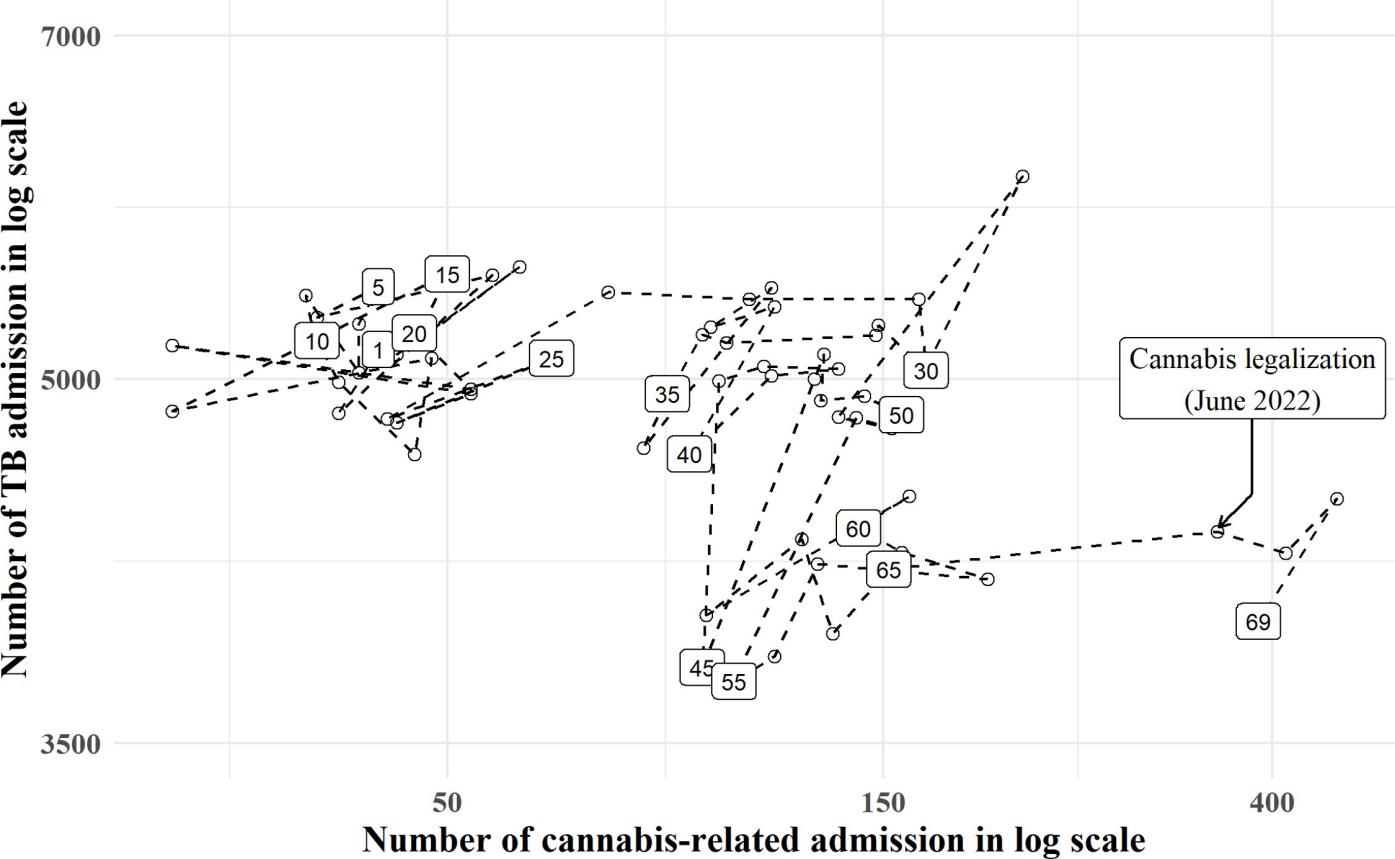
Chronological line plot of admission frequencies of cannabis-related and pulmonary TB diagnoses. The X-axis denotes the number of cannabis-related cases and the Y axis denotes that of pulmonary TB admissions in the same month. The number in the points denotes the number of months of follow up: month 1 denotes January 2017 and month 5 denotes May 2017, stepping up at 5-month intervals until month 69, denoting September 2022.

**Fig 1** illustrates the dynamics of monthly admissions from cannabis-related causes and those from pulmonary TB. From month 1 to 27, the frequency of cannabis-related admissions was relatively low (below 55 admissions per month), whereas that of TB admission was relatively high (above 4500 admissions per month). During the coronavirus disease 2019 (COVID-19) pandemic, from months 28 to 65, the number of monthly cannabis-related admissions was distributed along the middle of the X axis (ranging from 82 to 213 admission per month), while pulmonary TB admissions were widely distributed on the Y axis. Finally, during months 66 to 69, the number of cannabis-related admissions reached its peak, in contrast to that of pulmonary TB admissions.

**Fig 2:**
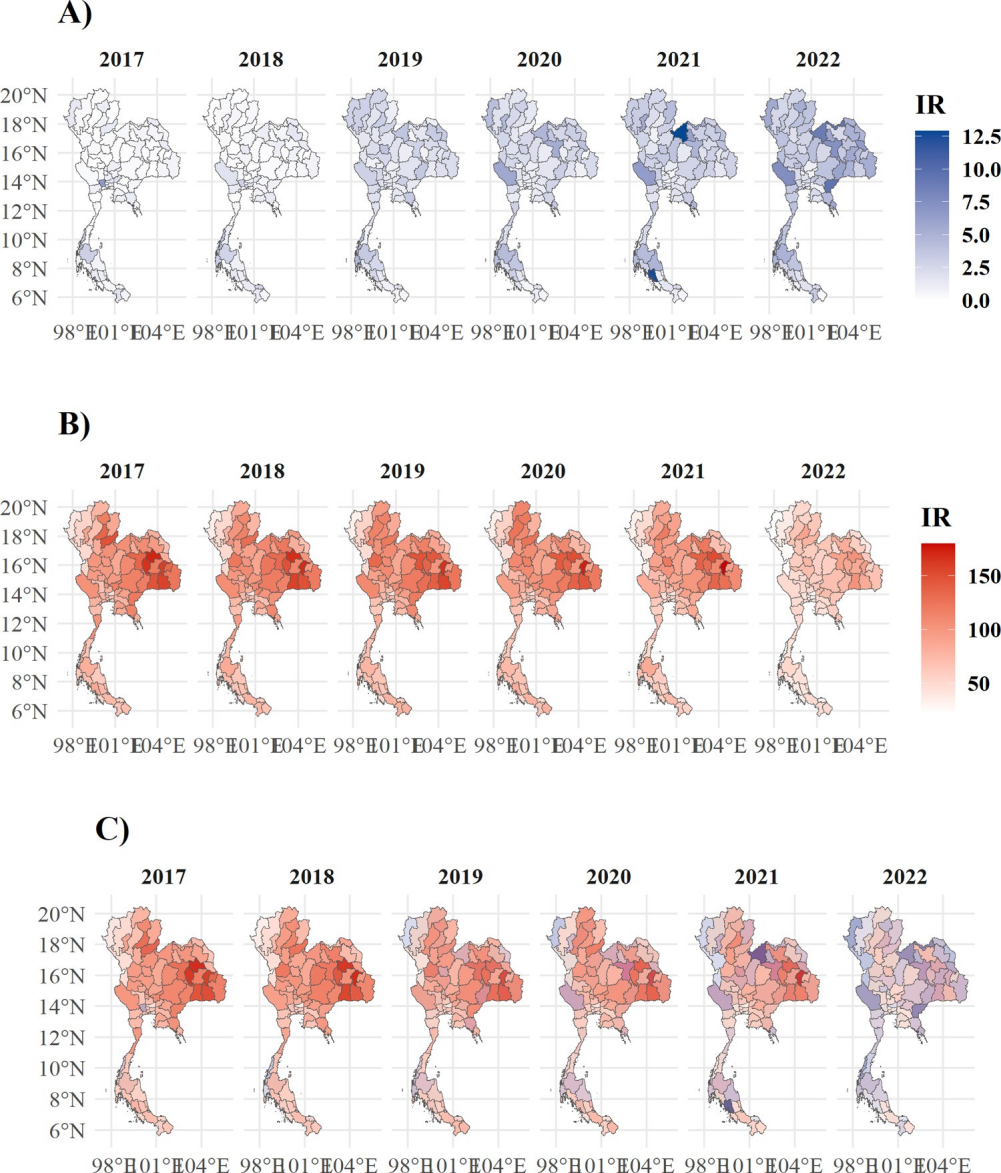
Thailand choropleth map based on the admission incidence rate per 100,000 population. A) cannabis-related causes, B) pulmonary TB, and C) the combination of both gradients in blue, red, and combined tones, respectively. Shapefile from GADM database (https://gadm.org) was used as the map’s polygon under a CC-BY license. The map was created using the R program version 4.2.3 [24].

**Fig 2** illustrates the spatiotemporal changes of IR of cannabis-related admissions (**2A**), TB admissions (**2B**), and their combination (**2C**). Distinct increases in cannabis-related admissions were observed in the northern regions of Thailand, eventually overtaking the initial relatively high IR in Surat Thani province in the middle of Southern Thailand. Conversely, TB admission incidence remained the highest in the Northeastern region throughout the years, although the incidence tended to decline over time in all regions.

### Matched cohort results

**Fig 3:**
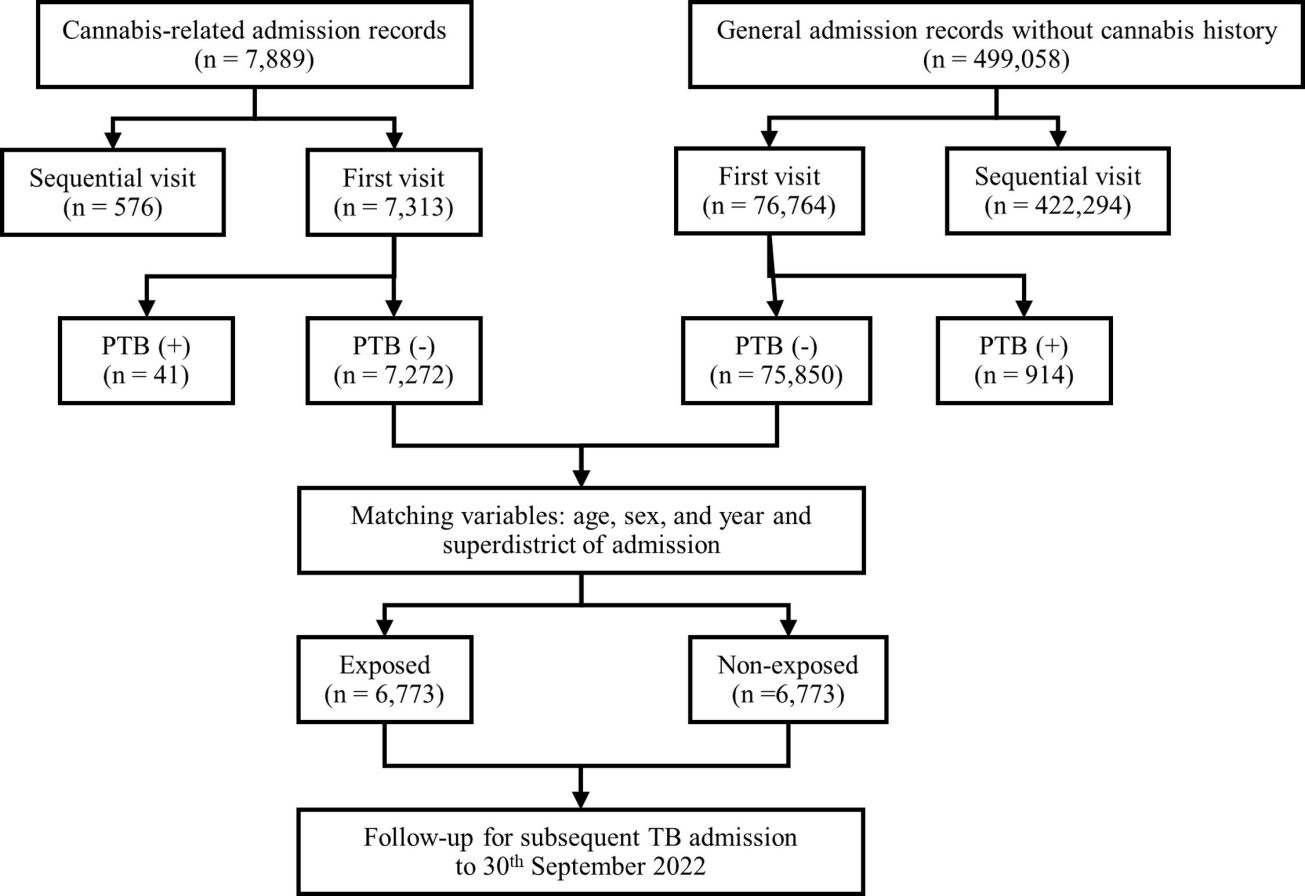
Flow diagram of data curation and matching process.

The flow diagram of data curation and cohort matching processes is shown in **Fig 3**. Throughout the 6-year period, 7,313 in-patients were admitted with cannabis-related causes recorded in their first data entry. The most common cannabis-related causes were “Mental and behavioral disorders due to use of cannabinoids” (83.5%) and “Poisoning, cannabis (derivatives)” (23%), with the diagnoses not being mutually exclusive. After excluding those co-diagnosed with pulmonary TB, 7,272 in-patients remained. Of these, 80 (1.1%) in-patients were deceased at the time of their first admission records.

We identified 37 subsequent pulmonary TB admissions among 23 in-patients, with 6 of these in-patients having more than one sequential TB admission. More than half (52.1%) of the 23 in-patients were admitted for TB within 10 months of their initial cannabis-related admission. Among the 23 in-patients, 78.26% were diagnosed with “Mental and behavioral disorders due to use of cannabinoids” on first admission, while “Poisoning, cannabis (derivatives)” was found in 26.09% of the patients. Other co-diagnoses occurring in > 10% of these in-patients included “Mental and behavioral disorders due to use of alcohol” (17.39%), “Intentional self-poisoning by and exposure to narcotics and psychodysleptics” (13.04%), “Mental and behavioral disorders due to use of other stimulants, including caffeine” (17.39%), “Hypo-osmolality and hyponatremia” (17.39%), “Hypokalemia” (17.39%), “Pneumonia, unspecified” (13.04%), “Poisoning by and exposure to narcotics and psychodysleptics” (13.04%), and “Type 2 diabetes mellitus without complications” (13.04%).

For the control group, we obtained admission records of 75,850 general in-patients with no prior history of cannabis-related admission or co-diagnoses indicating pulmonary TB. Among these, 560 (0.7%) were deceased at the time of their first admission record. Regarding the matching of the two cohorts, we successfully matched 6,773 (93.1%) of the 7,272 in-patients diagnosed with cannabis-related causes with the general in-patients without a cannabis-related admission history or pulmonary TB in their first admission.

**Table 1** summarizes the background information of the cannabis-related admission cohort with their matched pairs. The distributions of age and sex were well-matched, with a pre-dominance of young adults and male sex. The control participants were less likely to have metabolic comorbidities, such as diabetes mellitus, hypo-osmolarity and hypokalemia, but were more likely to have pneumonia.

**Table 1:** Descriptive table of two cohorts.

Variable	No history of cannabis-related admission	Previously admitted from cannabis-related causes	*P*-value
Total	6773	6773	
Age			0.893^a^
Median (IQR)	30 (21,47)	30 (21,47)	
Sex			1^b^
Male	5746 (84.8)	5746 (84.8)	
Female	1027 (15.2)	1027 (15.2)	
Diabetes mellitus			< 0.001^b^
No	6542 (96.6)	6465 (95.5)	
Yes	231 (3.4)	308 (4.5)	
Hypo-osmolarity			< 0.001^b^
No	6610 (97.6)	6543 (96.6)	
Yes	163 (2.4)	230 (3.4)	
Hypokalemia			< 0.001^b^
No	6099 (90)	5650 (83.4)	
Yes	674 (10)	1123 (16.6)	
Pneumonia			< 0.001^b^
No	6651 (98.2)	6698 (98.9)	
Yes	122 (1.8)	75 (1.1)	

^a^Mann–Whitney U test, and

^b^Chi-squared test

**Table 2** presents various follow-up outcome variables in the univariate analysis format. The univariate analyses revealed a shorter interval between the first admission and the subsequent TB admission and higher IDR in the case group. However, none of these differences reached statistical significance (*P* < 0.05) in any of the analysis methods used.

**Table 2:** Comparison of variables related to pulmonary TB admission between the two cohorts.

Variable	No history of cannabis-related admission	Previously admitted for cannabis-related causes	*P-*value
Total	6773	6773	
Individual level			
Frequency of subsequent TB admission			0.392^a^
0	6760 (99.8)	6751 (99.7)	
1	8 (0.1)	16 (0.2)	
2	3 (0)	3 (0)	
3	1 (0)	0 (0)	
4	0 (0)	1 (0)	
5	1 (0)	2 (0)	
Time until first subsequent TB admission (in-month)			0.049^b^
Median (IQR)	4.1 (0.4–13.5)	11.4 (4.0–32.7)	
Population level			
Frequency of subsequent TB admission			0.087^c^
Frequency	22	36	
Follow-up person year			
Median (IQR)	1.6 (0.5,3.1)	1.7 (0.5,3.2)	
Incidence density rate per person year per 100,000 population			0.087^c^
IDR	165.9	267.6	

^a^Fisher’s exact test

^b^Mann–Whitney U test

^c^Exact Poisson test

**Table 3** summarizes the results from the Cox regression for subsequent TB admission, including the HR and its 95% CI. The HR of the cannabis cohort was not significant (1.48, 95% CI: 0.74,2.96). Young age (< 40 years old), having diabetes mellitus, hypo-osmolarity, and pneumonia in the first admission were statistically significant predictors for subsequent pulmonary TB admission.

**Table 3:** Hazard ratio table.

Variable	Hazard ratio	95% CI	*P-*value
History of prior cannabis-related admission (ref = no)			0.268
Yes	1.48	(0.74,2.96)	
Age group (ref = < 40 years old)			0.002
≥ 40 years old	3.45	(1.6,7.43)	
Sex (ref = female)			0.055
Male	4.11	(0.97,17.51)	
Diabetes mellitus (ref = no)			0.001
Yes	4.22	(1.77,10.08)	
Hypo-osmolarity (ref = no)			0.003
Yes	4.39	(1.67,11.53)	
Hypokalemia (ref = no)			0.561
Yes	0.76	(0.29,1.94)	
Pneumonia (ref = no)			< 0.001
Yes	10.12	(3.92,26.12)	

## Discussion

While the monthly frequencies of pulmonary TB admissions declined, those that were cannabis-related increased from 2017 to 2022. The increase in cannabis-related admissions was classified into three time periods. From January 2017 to March 2019, the monthly admissions ranged from 25 to 75 nationally. From April 2019 to May 2022, the monthly admissions ranged from 82 to 213 nationally. In June 2022, recreational cannabis was legalized; the number of admissions then further increased to over 348 national monthly admissions until the last data point available in September of 2022. Both cannabis and TB admissions shared a geographical hotspot in the Northeastern region.

In the analysis of anonymized in-patients, the risk of subsequent TB admission among those previously admitted for cannabis-related issues was not significantly higher than that for those admitted due to other causes or diseases.

The steady decline in TB admissions during the pre-COVID-19 period may partially reflect the success of the National End TB Program [25]. The sharp decline during the COVID-19 period, however, is likely attributable to the disruption of the health care system from the pandemic. Similar declines in notified cases were reported in China [26, 27], the USA [28], and several lower-and middle income countries [1]. However, rebounds in notified cases after the pandemic subsided was also detected in several regions, including the Americas and South-East Asia [1]. In addition, several reports showed an increase in TB mortality while the notification rate declined during the pandemic [26, 29, 30].

Our data suggest that legalization of recreational cannabis may increase the incidence of hospitalization from cannabis usage. Availability of cannabis outlets was shown to lead to a higher likelihood of cannabis consumption [31], thereby increasing the chance of hospital admission. Similar findings of increased cannabis consumption after legalization have previously been reported; Martins et al. detected an increase in past-year cannabis use of 1–3.7% in the US [32]. Following the legalization of cannabis in Canada, Sandhu et al. reported that 20% of Canadians evinced an increased interest in trying cannabis [33], and the prevalence of cannabis usage increased by 5.6% [34]. Similarly, increased hospitalization rates due to cannabis were also reported in Canada after cannabis commercialization during the pandemic [35].

Both TB and cannabis-related admissions were highest in the Northeastern region of Thailand, which is well-known for its poverty and general ill health [36, 37]. These factors may potentially explain the geographical association [38, 39]. However, the opposite trend suggests a non-causal association between cannabis use and TB admissions.

When analyzing individual-level associations, we did not detect an increased risk of subsequent TB admission in patients priorly admitted for cannabis-related causes. Therefore, this study does not provide evidence to support cannabis consumption as a risk factor for pulmonary TB. In a non-hospitalized context, a contact-tracing study in Queensland [18], and a case-control study in Thailand reported an increased TB risk associated with cannabis use only among individuals who shared bong or water pipes, but not among oral cannabis consumers and non-sharing cannabis smokers [40]. Thus, so far, only bong sharing has been identified as a risk factor for TB transmission. Hypotheses related to respiratory tract damage causing depletion of local immunity remain unsupported by evidence. However, the lack of association found in this study does not provide sufficient grounds for policy implications regarding cannabis regulation, given the need to consider other adverse health effects of cannabis that are beyond the scope of this investigation.

The limitations of this study stemmed from the usage of a national in-patient data registry. We could not rule out possible past TB status; thus, the outcomes in this study were either new TB (either from new infection or from reactivation of latent TB) or relapse TB cases, with symptoms severe enough to require hospitalization. Patients in cannabis-related admission cases may exhibit different settings and behaviors compared to the general cannabis consumer population. A previous study show that bong sharing was a significant risk factor of active TB whereas oral consumption or even smoking with sharing bong were not. [40] In the current study, the use of hospitalization data based on ICD-10 codes limited us in acquiring information regarding route, type, duration, and frequency of cannabis consumption. This information is vital when assessing associations between infection and substance usage, as the pathogenesis can vary depending on how the substance was consumed. Similar limitations were also highlighted in another study investigating the impact of cannabis consumption on primary spontaneous pneumothorax [41]. These limitations should be corrected in the future studies.

## Conclusion

Both spatiotemporal and cohort analyses in this study failed to support the hypothesis that cannabis consumption increased pulmonary TB risk. Other study types are needed to further assess the association.

## Supporting information

S1 Dataset ICD-10 codes used for query process. (CSV)

S2 Dataset Monthly aggregated data used for Fig 1. (XLSX)

S3 Dataset Spatial polygon data used for Fig 2. (ZIP)

S4 Dataset Diagnosis frequency. (XLSX)

S5 Dataset Individual-level data of matched cohort results. (XLSX)
